# A New Lebanese Medication Adherence Scale: Validation in Lebanese Hypertensive Adults

**DOI:** 10.1155/2018/3934296

**Published:** 2018-05-20

**Authors:** R. Bou Serhal, P. Salameh, N. Wakim, C. Issa, B. Kassem, L. Abou Jaoude, N. Saleh

**Affiliations:** ^1^Department of Epidemiology and biostatistics, Faculty of Public Health, Lebanese University, Fanar, Lebanon; ^2^Faculty of Pharmacy, Lebanese University, Al Hadath, Lebanon; ^3^Department of Nutrition and Dietetics, Faculty of Public Health, Lebanese University, Fanar, Lebanon

## Abstract

**Background:**

A new Lebanese scale measuring medication adherence considered socioeconomic and cultural factors not taken into account by the eight-item Morisky Medication Adherence Scale (MMAS-8).* Objectives* were to validate the new adherence scale and its prediction of hypertension control, compared to MMAS-8, and to assess adherence rates and factors.

**Methodology:**

A cross-sectional study, including 405 patients, was performed in outpatient cardiology clinics of three hospitals in Beirut. Blood pressure was measured, a questionnaire filled, and sodium intake estimated by a urine test. Logistic regression defined predictors of hypertension control and adherence.

**Results:**

54.9% had controlled hypertension. 82.4% were adherent by the new scale, which showed good internal consistency, adequate questions (KMO coefficient = 0.743), and four factors. It predicted hypertension control (OR = 1.217; *p* value = 0.003), unlike MMAS-8, but the scores were correlated (ICC average measure = 0.651; *p* value < 0.001). Stress and smoking predicted nonadherence.

**Conclusion:**

This study elaborated a validated, practical, and useful tool measuring adherence to medications in Lebanese hypertensive patients.

## 1. Introduction

Hypertension is an international public health burden that caused around 9,4 million deaths in 2010 [1]. In Lebanon, hypertension prevalence was 36,9% in adults aged 21 years and above [2]. Reducing sodium intake could significantly lower blood pressure in hypertensive and normotensive patients [3], as well as diminishing the risk of stroke and fatal coronary artery diseases in adults [4]. A wide variety of efficient medications to control hypertension is available [5]. Yet, low rates of controlled hypertension were observed. In USA, 47% of all hypertensive patients and 60% of treated hypertensive patients had controlled blood pressure [6], compared to, respectively, 27% [2] and 48.9% [7] in Lebanon. Patients with uncontrolled blood pressure had a higher risk of nonadherence to their medication [8], while adherent patients had lower systolic blood pressure and diastolic blood pressure [9].

Medication adherence was defined by the WHO as “the extent to which a person's behavior, taking medication, following a diet, and/or executing lifestyle changes, corresponds to agreed recommendations from a healthcare provider” [10]. In Lebanon, 22.4% of hypertensive patients were nonadherent to their medication [11]. Adherence amelioration was important to reduce hospitalization and mortality due to cardiovascular diseases [12], as well as resulting costs [13]. A precise evaluation of medication adherence and the factors that affect it is needed, with adequate tools to be used in routine clinical practice [14]. The eight-item Morisky Medication Adherence Scale (MMAS-8) is a subjective method, with a 93% sensitivity and a 53% specificity [15]. Even though it is frequently used in studies, as it is simple and not expensive, MMAS-8 does not take patients' cultural and socioeconomic factors into account, as well as their relationship with their healthcare providers, while these variables affect adherence. In this context, a new scale (Lebanese Medication Adherence Scale: LMAS) was developed, taking into account factors related to the Lebanese culture. It evaluated psychological, occupational, economical, and annoyance factors.

The primary objective of this study was to validate the LMAS in Lebanese hypertensive adults, to assess its ability to predict hypertension control, and to compare it with MMAS-8. The secondary objective was to evaluate the adherence rate in Lebanese hypertensive adults, and to determine factors affecting antihypertensive adherence.

## 2. Methodology

### 2.1. Score Creation

The new score (LMAS) was inspired by the Morisky score, adding other variables affecting adherence, discussed by the investigators with physicians and community pharmacists in a focus group. The reasons why MMAS-8 was our source of inspiration were its simplicity and validity, as well as its common use among clinicians and researchers (in Lebanon and elsewhere) to asses antihypertensives adherence. MMAS-8 contains seven questions with dichotomic answer (yes/no) and one Likert scale question with five possibilities and measures forgetfulness, medication taking behavior, and secondary effects [16]. The Arabic version of MMAS-8 had already been validated in Lebanon [17].

LMAS assessed forgetfulness by a question evaluating its frequency, inspired by merging MMAS-8's questions regarding forgetfulness which investigate the past day, the past two weeks, and the general forgetting behavior. Moreover, a question regarding the frequency of reminders' (by caregivers, alarms, etc.) use to increase adherence was added, knowing that social interactions take a privileged place in the Lebanese culture and that reminders might affect adherence. Also inspired from MMAS-8, we included questions evaluating adherence change if the patients felt clinically better or worse, but behavior change if laboratory exams improved was added to LMAS. Furthermore, adherence when away from home was included in the new score, with an adaptation by adding suggestions of outdoor diner or lunch. A question regarding adherence during busy periods was also included in LMAS. In addition, economic factors, like adherence when health coverage does not pay for the medicine and delay in buying a new pills box when the old one becomes empty, were included in LMAS. Besides, adherence behaviors in case of boredom, if some food items were prohibited during treatment period, or if the patient or his neighbor (because these issues could be discussed often between Lebanese patients) experienced any secondary effects, were evaluated. Likewise, adherence behavior when the patients noticed that they took a lot of pills was tested. Consequently, LMAS contained 16 Likert scale questions with four options to answer each (coded from zero (less adherence) to three (higher adherence)) and was subject to validation.

### 2.2. Study Design, Procedure, Inclusion Criteria, and Sample Size

An observational cross-sectional study was conducted. Patients were recruited from the outpatient cardiology clinics of three hospitals in Beirut, visited at random dates in 2016, during patients' appointments. Patients matching the inclusion criteria were invited to participate in the study, after signing a written informed consent. Their blood pressure was measured twice, at a 10-minute interval, with their right hand placed at the level of the heart [18], with the validated digital sphygmomanometer “Omron M3 IT Intellisense” [19]. To reduce white coat effect, the surveyor measuring patients' blood pressure was not wearing any gown, and the measurement was done in the waiting area before the patient enters to the clinic. Controlled hypertension was defined by blood pressure lower than 130/80 mmHg for diabetic patients and lower than 140/90 mmHg for the others [20]. Patients also filled a questionnaire, and a fresh urine sample was collected, labelled by a code, preserved in a refrigerated box, and carried on the same day to the laboratory for analysis.

The INTERSALT equation was used to assess mean sodium intake of treated Lebanese hypertensive patients, as this equation had less bias [21]. To reduce cost, the INTERSALT equation was calculated in a group of patients for whom the measures of urinary sodium, potassium, and creatinine were taken. Only urinary sodium was measured for the rest of the patients, and the value of the INTERSALT equation was estimated by a statistical analysis using linear regression. Correlation between the two measures of INTERSALT (estimated and calculated) was determined by intraclass correlation coefficient. Mean daily sodium intake of hypertensive patients was then obtained based on the estimated values of INTERSALT equation.

Patients included were Lebanese, aged 18 years and above, with primary hypertension diagnosed at least six months ago, treated with antihypertensives for at least the past six weeks, and having signed the informed consent. Exclusion criteria were secondary hypertension, pregnant women, being hospitalized, dement, or mentally disabled, and patients with physical disability or any infection affecting blood pressure.

A ratio of 20 : 1 (number of subjects : number of items) was used to calculate sample size [22]. This corresponded to 320 patients for the 16 questions of the LMAS. 405 patients were included, to account for a maximum of 20% missing answers or incomplete questionnaires.

### 2.3. Variables Included in the Questionnaire

Sociodemographic characteristics of the patients were assessed. Lifestyle characteristics included BMI, physical activity (in minutes/week), stress level during the last three months, smoking status and amount (calculated in year-packs for cigarettes and shisha [23]), caffeine consumption (in g/day based on USDA database [24]), alcohol consumption (number of glasses consumed), diet type, and autoevaluation of salt consumption. Information concerning hypertension, as disease duration, baseline blood pressure, medication, comorbidities (including diabetes, high blood lipid levels, cardiovascular diseases, and psychiatric disorders), frequency of physician visits, secondary effects, and MMAS-8 and LMAS scores, was also collected.

### 2.4. Statistical Analysis

We used SPSS version 20 for data entry and analysis. To validate LMAS, an exploratory factor analysis with Varimax rotation was done, leading to the reduced dimension score (LMAS-14). Internal consistency of LMAS-14 was assessed using Cronbach alpha, which showed good internal consistency when it was >0.5. Sensitivity and specificity were shown by ROC curve, with hypertension control as a reference variable. Cut-off value was determined by Youden Index, defined as *J* = maximum (sensibility + specificity − 1). [25]. Logistic regression (backward LR method), with hypertension control as a dependent variable, was used to determine which score was a better predictor of hypertension control. Concordance between adherence measures of the two scores was evaluated by the intraclass correlation coefficient (considered good when it was >0.5).

To determine the factors affecting medication adherence, the dependent variable was adherence measured by LMAS-14. Each variable with *p* value ≤ 0.2 in bivariate analysis was included in the logistic regression (backward LR method). A cut-off of 6 was admitted to classify patients as adherent (MMAS-8 score between 6 and 8) and nonadherent (MMAS-8 score < 6). A cut-off value for classifying patients for the LMAS-14 was determined by ROC curve and Youden Index.

## 3. Results

### 3.1. Descriptive Statistics

Patients' mean age was 65 years; almost half (52.2%) were females and the majority resided in Beirut (47.2%). 12.9% had no health coverage. As for lifestyle, almost half of patients (42.2%) had a moderate stress level (Table 1).

Mean blood pressure measured was 132.94/80.75 (±15.36/12.02) mmHg. The majority of patients (54.9%) had controlled blood pressure. MMAS-8 showed that 86.1% of the patients were adherent to antihypertensive pills, while adherence rate using the LMAS-14 was 82.4% (Table 2).

### 3.2. LMAS Score Validation

#### 3.2.1. Factor Analysis

Factor analysis was used to reduce the number of the score's questions and to study its principal components. After rotation, two questions were removed, as the correlation matrix showed they had correlation coefficients < 0.3 with other questions, and one of them had an anti-image correlation coefficient of 0.38 (<0.5). The new score, consisting of 14 questions, had a KMO coefficient of 0.743, showing adequate questions. The Bartlett test was significant (*p* value < 0.001), proving that the model was good. Anti-image correlation matrix showed positive values in the diagonals, proving that the questions were correlated; the majority of the values were above 0.8. In the principal component analysis, the used model explained 58.259% of the variance of the score's answers. Factor analysis showed a scale of four factors, with load > 0.4 (Table 3): the first (Cronbach alpha = 0.695.) included occupational factors that encouraged forgetting or stopping medication and consisted of five questions. The second, psychological factor (Cronbach alpha = 0.591), consisted of four questions. The third, annoyance factor (Cronbach alpha = 0.48), had three questions. Finally, the economical factor (Cronbach alpha = 0.48) consisted of two questions (Table 3).

#### 3.2.2. Sensitivity, Specificity, and Cut-Off Value of LMAS-14

A cut-off value of 38 was determined for the LMAS-14, with a sensitivity of 82.9% and a specificity of 36.9%.

#### 3.2.3. Prediction of Hypertension Control

*(1) Daily Sodium Intake Estimation. *Intraclass correlation coefficient between the variables was created by the estimation of INTERSALT (using urinary sodium measure) and the calculated INTERSALT was 0.807 for average measures (CI = [0.607–0.905]; *p* value < 0.001), proving a good correlation. We used the new variable in the following analysis. Mean daily sodium intake was 3.48 (±0.78) grams in hypertensive patients. No statistically significant difference in mean sodium intake (*p* value = 0.827 for Student's *t*-test) was found between patients declaring to follow a low salt diet (mean = 3.47 g/day) and those denying it (mean = 3.5 g/day).

*(2) Predictors of Hypertension Control. *Patients having a higher LMAS score had an OR of 1.217 (*p* value = 0.003) of achieving controlled blood pressure. Thus, having a longer disease duration (OR = 0.914; *p* value = 0.014) and a higher number of comorbidities (OR = 0.643; *p* value = 0.044) predicted lower hypertension control (Table 4).

#### 3.2.4. Concordance between LMAS-14 and MMAS-8

Intraclass correlation coefficient showed good concordance between MMAS-8 and LMAS-14 scales (ICC average measure = 0.651; *p* value < 0.001).

### 3.3. Factors Affecting Adherence

A higher stress level (OR = 0.542; *p* value = 0.007) and being a past or actual smoker (OR = 0.506; *p* value = 0.013) decreased the probability of being adherent to antihypertensives by LMAS-14 (Table 5).

## 4. Discussion

Factor analysis allowed the selection of 14 questions divided in four factors for the LMAS-14. This is in accordance with the study that created the score by elaborating a scale divided in four factors [26]. Hypertension control rate was higher than other Lebanese studies in treated hypertensive patients [2, 7]. This could be due to recruiting study participants from outpatient clinics in Beirut hospitals, so these patients might have a better follow-up than the general population. Thus, results are lower than those (62.9%) found in a study at Beirut hospitals [11], probably due to the consideration of a lower cut-off for diabetic patients. Other countries had lower hypertension control rates [6, 22, 27–29].

Association between adherence to medication and hypertension control has already been demonstrated [7, 9]. LMAS-14 seems to be a better predictor of hypertension control in the Lebanese population; this could be due to taking into consideration the psychological and cultural factors. The good concordance between the two scores could be explained by the fact that LMAS-14 was inspired from MMAS-8 and both measure adherence. A higher number of comorbidities predicted lower hypertension control. This could be explained by the necessity, for patients with more comorbidities, to have a more complex treatment regimen with a wider variety of medication and thus to be less adherent to their medication and have lower blood pressure control. Having longer disease duration was a predictor of lower hypertension control, matching the findings of Sarfo et al. [30].

The mean sodium intake of our population was around 3.48 g/day, while a significant blood pressure decrease was observed with a mean sodium intake below 2 g/day [4]. The absence of statistically significant difference in mean daily sodium consumption, between patients declaring to follow a low sodium diet and those who do not, suggests either a social desirability bias or the inefficiency of those regimes.

This study showed higher adherence rates for LMAS-14 (82.4%) than another one in Beirut (77.6%) [11]. This could be due to a potential social desirability bias or due to the recruitment from hospitals clinics. Higher stress level predicted nonadherence to LMAS-14, concordant with a study in Beirut showing that anxiety reduced adherence [17].

Study strengths include sample size and control for sociodemographic, lifestyle, hypertension, comorbidities, and adherence characteristics. To our knowledge, it is the first study in Lebanon that estimated daily sodium consumption and adjusted hypertension control predictors to it. We also obtained a validated scale measuring adherence, with good psychometric properties, concordant with Morisky score, and predicting hypertension control. Factors affecting adherence were also defined and were concordant with other studies.

However, some limitations are to be mentioned. A potential selection bias could be due to the recruitment done in cardiology outpatient clinics of three hospitals in Beirut. An overrepresentation of the Lebanese residing in Beirut and Mount Lebanon was also noted. A potential information bias could be due to the employment of subjective methods to measure adherence, but objective ones are expensive and are not available in Lebanon. Social desirability bias could have modified the classification of adherence in patients and its association with hypertension control. White coat effect was only prevented by the surveyors' dress code excluding hospital gowns and by the measurement technique, done outside the doctor's office in the waiting area, but baseline blood pressure before presentation to the clinic was not obtained, for the majority of the patients, due to the unavailability of sphygmomanometers at their house. Finally, financial constraints did not allow the measurement of all the necessary parameters for INTERSALT calculation to all patients; consequently, its estimation was done in the majority of patients who gave spot urine samples.

## 5. Conclusion

This study elaborated a validated, practical, and useful tool measuring adherence to medications in Lebanese hypertensive patients. Promotion of adherence to antihypertensives by physicians and public health actors is advantageous, as well as better stress management in Lebanese hypertensive patients. Further research is needed to estimate mean sodium intake in hypertensive patients and the consistence of their diets. A comparison between LMAS-14 and MMAS-8 using an objective measure of adherence might also be beneficial for future studies.

## Figures and Tables

**Table 1:** Sociodemographic and lifestyle characteristics.

Variable	Items	Mean/*N*	SD/%
Age		65.05	12.93

Sex	Male	197	48.8
	Female	207	51.2

Place of residence	Beirut	191	47.2
	Mount Lebanon	165	40.7
	North	4	1
	South	29	7.2
	Bekaa	16	4

Educational level	Illiterate	41	10.4
	Primary	101	25.7
	Complementary	81	20.6
	Secondary	68	17.3
	University	102	26

Frequency of physician's consultation	No routine consultation	131	32.9
	Once in six months	79	19.8
	Once in three months	151	37.9
	Once a month	32	8
	Once or more every two weeks	5	1.3

Health coverage	Self-payer	52	12.9
	Social security	132	32.7
	Cooperative of state employees	37	9.2
	Army	2	0.5
	General security	8	2
	Private insurance	121	30
	Nongovernmental organization	36	8.9
	Other	16	4

Socioeconomic status (crowding index)	Low socioeconomic status	9	3.6
	Moderate	111	44.8
	High	128	51.6

BMI	Underweight	2	0.5
	Normal	107	26.7
	Overweight	159	39.7
	Obese	133	33.2

Physical activity	No physical activity	285	71.1
	Lower than 150 min/week	24	6.0
	150 min/week or higher	92	22.9

Smoking	Never	199	49.1
	Stopped	78	19.3
	Still smoking	128	31.6

Stress level	Very low	10	4
	Low	49	19.7
	Moderate	105	42.2
	High	48	19.3
	Very high	37	14.9

Caffeine intake per day (mg)		67.67	53.17

Low sodium diet	No	143	36.6
	Yes	248	63.4

**Table 2:** Hypertension characteristics.

Variable	Items	Mean/*N*	SD/%
Mean systolic blood pressure		132.94	15.36
Mean diastolic blood pressure		80.75	12.02
Disease duration		9.45	7.05
Number of antihypertensive pills per day		1.97	1.06
Total number of pills per day		5.16	2.99
Comorbidities	No	127	31.7
	Yes	274	68.3
Secondary effects	None	240	60.5
	At least one	157	39.5
Hypertension control	Uncontrolled hypertension	141	45.1
	Controlled hypertension	263	54.9
MMAS-8	Nonadherent	56	13.9
	Adherent	348	86.1
LMAS-14	Nonadherent	71	17.6
	Adherent	333	82.4

**Table 3:** Questions, factors, and their internal consistency.

Factor	Cronbach alpha	Load	Question
Occupational	0.695	0.771	Do you forget to take your medication when you are busy (intensive work or travel)?
		0.722	Do you forget to take your medication if you are invited to lunch or dinner?
		0.712	Do you forget to take your medication?
		0.526	Do you get late when it comes to buying your medication packs when they become empty?
		0.438	Do you stop taking your medication if it forbids you from eating certain food that you love because of possible food-medication interaction?

Psychological	0.591	0.832	Will you stop taking your medication, without your doctor's consultation, if your neighbor/relative took a prescription like yours for a long term and it caused them side effects?
		0.709	Do you stop taking your medication without consulting your doctor if the laboratory tests show improvement during treatment period?
		0.685	Do you stop taking your medication without consulting your doctor if you do not feel better during treatment period?
		0.521	Do you stop taking your medication without consulting your doctor if you feel better during treatment period?

Annoyance	0.48	0.890	Do you decide to stop some of your medications without consulting your doctor if you noticed that you are taking too many medications every day?
		0.701	Do you stop your chronic treatment if you get bored of it?
		0.502	Do you stop taking your medication in case of side effects?

Economical	0.679	0.856	Do you stop taking your medication if your insurance does not cover it?
		0.833	Will you stop buying your medication packs if you considered them expensive?

Extraction method: principal component analysis. Rotation method: varimax with Kaiser normalization.

**Table 4:** Logistic regression for hypertension control.

Variables	Exp (*B*)	95% CI		*p* value
Physical activity duration	0.998	0.996	1.000	0.063
Disease duration	0.914	0.851	0.982	0.014
Number of comorbidities	0.643	0.418	0.989	0.044
LMAS-14	1.217	1.069	1.386	0.003

Omnibus test *p* value < 0.001; Hosmer-Lemeshow test *p* value = 0.491; Nagelkerke's *R*^2^ = 0.215; overall predicted percentage = 69.6%. *Independent variables*: age, physical activity duration, disease duration, number of comorbidities, number of secondary effects symptoms, BMI, MMAS-8, LMAS_14, estimated INTERSALT, educational level, frequency of physician's consultation, and health coverage.

**Table 5:** Logistic regression for LMAS-14.

Variables	OR	95% CI		*p* value
Stress level	0.542	0.348	0.845	0.007
Smoking	0.506	0.297	0.864	0.013

Omnibus test *p* value < 0.001; Hosmer-Lemeshow test *p* value = 0.760; Nagelkerke's *R*^2^ = 0.185; overall predicted percentage = 72.2%. *Independent variables*: age, number of antihypertensive pills, number of secondary effects, BMI, employment, adding salt to food at the table, food evaluation, frequency of physician's consultation, stress level, health coverage, crowding index, smoking, and estimated INTERSALT.

## Abbreviations

MMAS-8: Eight-item Morisky Medication Adherence Scale
LMAS-14: Fourteen-item Lebanese Medication Adherence Scale.
